# Assessing the Use of Welfare Technology in Social Care for Older Adults Through Assistant Nurses’ Perceptions of Upskilling and Care Delivery Outcomes: Cross-Sectional Study

**DOI:** 10.2196/65641

**Published:** 2025-08-26

**Authors:** Mahwish Naseer, Lotta Dellve

**Affiliations:** 1 Department of Sociology and Work Science, University of Gothenburg, Skanstorget 18, Gothenburg, SE 41122, Sweden, 46 0317861391

**Keywords:** effects of technology in social care, care users, skill development, training, assistant nurses, care providers, digitalization, older adults, geriatric, care, continuity of care, social care, loneliness, older adult care

## Abstract

**Background:**

The implementation of welfare technologies, a broad array of technologies that have the potential to maintain or improve individuals’ safety, independence, and participation, has increased rapidly in recent years, offering new ways of delivering care. However, studies of welfare technology use in the social care sector are scarce.

**Objective:**

This study aims to explore the use of different types of welfare technologies, training in the use of these technologies, and to identify their associations with outcomes for care recipients and frontline care workers in the social care of older adults.

**Methods:**

A cross-sectional survey was conducted based on a nationwide randomized sample of assistant nurses employed in social care for older adults in Sweden (N=1163; response rate 23%). Dependent variables were outcomes for care recipients (continuity of care, participation, and reduction in loneliness) and upskilling for frontline care workers. The exposure variables were types of welfare technologies and training in the use of such technologies. Associations between exposure and dependent variables were assessed through logistic regression models.

**Results:**

According to the perceptions of care workers, interactive technologies were significantly positively associated with continuity of care (odds ratio [OR] 1.58, 95% CI 1.15‐2.18), participation (OR 2.01, 95% CI 1.48‐2.74), and reduction in loneliness among care recipients (OR 1.92, 95% CI 1.41‐2.62). In addition, there was a significant positive association between interactive technologies and upskilling of care workers (OR 2.44, 95% CI 1.58‐3.79). Despite the benefits of welfare technology, the effects can also be negative, as shown by the findings on the use of digital documentation (OR 0.69, 95% CI 0.49‐0.96), digital locks or cameras or sensors (OR 0.62, 95% CI 0.46‐0.84), and the lower likelihood of participation. Training in the use of welfare technology was significantly associated with outcomes for care recipients (continuity of care: OR 2.02, 95% CI 1.53‐2.66; participation: OR 1.91, 95% CI 1.45‐2.51; reduction in loneliness: OR 1.74, 95% CI 1.31‐2.30), as well as upskilling of care workers (OR 4.59, 95% CI 3.28‐6.42). The interaction analyses showed that participants who had not received any training but used digital documentation reported favorable views on continuity of care and upskilling, whereas those who had received training expressed concerns about participation and addressing loneliness.

**Conclusions:**

The potential outcomes of welfare technology use in social care for older adults can vary with the types of technologies used. Care workers hold positive perceptions toward interactive technologies to improve care delivery outcomes and skill development. The findings on training and improved outcomes suggest that investing resources to promote training can reinforce positive experiences. While training had a positive impact, the interaction effects of digital documentation and training were mixed.

## Introduction

### Background

The use of digital services has increased rapidly in recent years to address health care and social welfare challenges [1], such as the care needs of aging populations and the shortage of frontline care workers [2]. Welfare technology, a term used in the Nordic context, covers a broad array of technologies that have the potential to maintain or improve individuals’ safety, independence, and participation, thereby promoting their quality of life [3,4]. The efficient use of care workforce resources is another expected outcome of welfare technology; for example, automation reduces the need for face-to-face appointments for minor issues and frees up resources for tasks that really require human contact [4,5]. Hence, new ways of delivering both traditional and emerging types of welfare services offer potential benefits for care recipients as well as care workers [2]. Due to these constant changes, up-to-date knowledge is needed about the effects of digitalization on social care practices. In Sweden, assistant nurses are the main workforce providing social care for older adults. For the sustainable deployment of technology, it is essential that these frontline workers perceive its use as beneficial both for themselves and for their care recipients. In this study, we explored associations between welfare technologies and potential benefits for care recipients (eg, continuity of care, participation, and lower levels of loneliness) and frontline care workers (eg, upskilling).

### Theoretical Perspectives

The perceived usefulness is an important component of theoretical models on technology acceptance in voluntary or mandatory settings [6-8]. It refers to the extent to which an individual believes that using technology enhances work performance. In social and health care contexts, the usefulness of welfare technology includes work performance as well as quality of care [8-11], which are broad and interrelated concepts. For instance, technology can improve workflow efficiency and simultaneously enhance care delivery, thereby influencing both aspects.

In the context of social care for older adults, continuity of care can be defined as the degree to which a series of discrete care events are perceived as coherent, connected, and consistent with the needs of older adults and personal context [12,13]. Digital care records, an example of welfare technology, provide quick access to the updated information, often entered by multiple care providers. This minimizes the risk of errors and enables timely referrals, thereby facilitating continuity of care [14], which is an indicator of quality of care. This implies that the types of welfare technologies contributing to continuity of care are perceived as useful.

Another indicator of perceived usefulness could be participation, an expected outcome of welfare technology in social care. Participation refers to the care recipients’ involvement in an area of life, such as social participation or being involved in care planning [2,3,15]. For example, if care recipients have access to their digital care records, they can participate in planning their own care [2,14].

Continuity of care and participation can be seen as instrumental indicators of quality of care. The perceived usefulness of welfare technology in addressing social issues such as loneliness provides a broader perspective. The risk of loneliness or social isolation increases with the advancement of age, owing to life situations emerging from health conditions or living arrangements. Although technology cannot replace face-to-face interactions, it can provide complementary functions that contribute to overcoming the barriers (eg, mobility challenges) of social interactions [16]. Thereby, technology may have the potential to address loneliness or social isolation among older adults.

The implementation of welfare technologies stipulates discussion on changes in work complexity and required skills. Work complexity encompasses nonrepetitive tasks, greater autonomy, and skill development [17]. Job designs with higher complexity levels present workers with new challenges and stimulate the acquisition of new skills, defined as upskilling [17,18]. The upskilling approach suggests that technology use has altered the occupational structure, leading to more complex work [17]. This implies a hypothesis on a positive association between the use of welfare technologies and the upskilling of frontline workers. However, a problem arises when the use of welfare technology is perceived to collide with professional identity [19]. Adequate training equips professionals with the skills required to use new technology and facilitates understanding of the justification for technology. These attributes of training contribute to positive experiences with, and the reinforcement of, technology use [9,11,20]. Consistent with previous research, we hypothesize that training is positively associated with upskilling and care delivery outcomes.

### Prior Research

A systematic review including studies on primary health care has shown mixed findings, which might be due to the multidimensional aspect of continuity of care [21]. Similarly, a study performed in Swedish primary health care has shown that an association between welfare technology and continuity of care could be understood in many ways [14]. For example, the use of digital records and communications increases the likelihood of informational continuity, whereas interrelation continuity (ie, between the patient and a single care provider) improves for some but not all patients [14,21].

A systematic review including studies on user feedback from older adults and their formal or informal caregivers has shown that welfare technologies can improve independence and self-management among older adults [10]. Here, independence refers to not depending on others for assistance [10], hence potentially improving the likelihood of participation in daily life activities [15]. According to the views of professionals in Swedish municipal social care for older adults, welfare technology could be an enabler of care recipients’ participation via access to their care plans [2]. Another systematic review has shown that technology contributes positively to improving social interaction between older adults and their formal or informal caregivers and to reducing loneliness among older adults [10]. Specifically, technology such as video calls and social media facilitated communication with others. However, interaction with robots is also appreciated by older adults for reducing loneliness [10]. During the pandemic, the majority of social care workers in a Welsh study perceived that technology benefited efforts to address loneliness or social isolation among older adults [16]. Despite receiving training in the use of technology and support in accessing it (eg, they could borrow tablets and have smart home devices and assistive technology such as Amazon Alexa installed), telephone calls remained the most common means of social interaction [16].

The level of education and occupational requirements are often used as broad measures of workforce skills [17]. However, using years of schooling as a proxy for skill has been criticized for its static nature. In contrast, job requirements are shaped by the nature of work tasks, which are dynamic and subject to change over time. Consequently, an individual’s ability to perform these tasks is influenced not only by formal education but also by prior experience and adaptability [17]. This underscores the need for continuous skill development to address the evolving complexities of work, particularly in the context of digitalization. Previous research has shown that digital competency is crucial to understanding the relevance of technology in clinical contexts in providing high-quality patient care and building positive experiences of technology use [1,20]. However, inadequate training in the use of technology may slow down the learning process [5].

In summary, there are high expectations regarding the potential benefits of using welfare technologies and providing training in their use. However, studies on the use of welfare technology in the social care sector are scarce, and therefore, there is limited empirical evidence to support that such expectations are being borne out [1]. This lack of empirical evidence on the desired benefits contributes to resistance to the deployment of technology [2]. Moreover, previous research has primarily focused on the potential benefits of a single type of welfare technology, such as digital communication [1,16,21]. A particular type of technology may have a positive effect on one indicator of quality of care but a negative effect on another. This suggests a need to understand the potential outcomes of different types of welfare technologies in the same context.

### Objective

This study aims to explore the use of different types of welfare technologies, training in the use of these technologies, and to identify their associations with outcomes for care recipients and frontline social care workers.

## Methods

### Study Design, Setting, and Population

A cross-sectional study design was used. In Swedish social care for older adults, assistant nurses make up the main workforce and comprise the population of this study. Assistant nurses aged ≥18 years and employed in social care for older adults in Sweden for a minimum of 5 years were selected through the Swedish Occupational Register. High turnover and short-term employment are common in social care for older adults. A 5-year experience requirement ensures that participants have adequate experience of social care of older adults to provide informed and critical perspectives on the use of welfare technology. A total of 70,696 eligible participants were identified. This study forms part of a larger project investigating the working environment of assistant nurses in social care of older adults, which is based on an earlier project examining working conditions in Swedish social care for older adults [22]. To enhance the potential for conducting subgroup statistical analyses, a representative sample size of 5000 participants was determined in collaboration with Statistics Sweden.

In Sweden, care for older adults is a public responsibility. A fundamental value in social care for older adults is to ensure that they live an independent, dignified life, including good mental health and well-being (5 chapter 4 § Social Services Act). The municipalities (N=290) are responsible for social care. Provision of basic home health care (up to nurse level) is also the responsibility of municipalities in all but one region. Both public and private providers can offer publicly funded social care. The implementation of welfare technology is shaped by a top-down approach. Local variations can be expected in the extent of deployment of welfare technologies due to the decentralized system. The sector of social care of older adults is characterized by a predominantly female workforce, and assistant nurses typically hold an upper secondary education.

### Data Collection and Sources

Data were collected from December 2023 to February 2024 through a survey questionnaire. The survey assessed working conditions in social care for older adults and included 46 items. It was adapted from a previous questionnaire on working conditions [22]; however, the dependent and exposure variables used in this study were newly developed. To construct items related to technology, training, and upskilling, the project team drew on the Swedish national report on eHealth and technology [23]. These items have been used in the national survey, thereby enhancing the likelihood of face validity.

Data collection was conducted in collaboration with Statistics Sweden and a research firm connected to the university. The population register was used to distribute invitations to potential participants’ postal addresses. Each participant received an invitation letter with study information, a web link, and a QR code providing access to the online questionnaire. To increase response rates, 2 reminder mailings were sent, including both a paper version of the questionnaire with a prepaid return envelope and the web link. The research firm administered the web-based survey using its proprietary platform and applied a template questionnaire structure tailored to question types and scales. Paper-based responses were scanned, and the data were transferred into the dataset using the research firm’s software. Quality assurance procedures included matching scanned responses with the original paper forms to ensure data accuracy.

In the next step, survey data were linked to the national register, the Longitudinal Integration Database for Health Insurance, and the Labor Market by Statistics Sweden to obtain data on sociodemographic variables.

### Ethical Considerations

Invited participants received information about the study and the confidentiality of their responses. Potential participants were informed that the survey would take approximately 30 minutes to complete, and that participation was voluntary, with the option to respond selectively to individual items. They were also informed that, upon accepting the invitation, their sociodemographic data would be obtained from national registers. Written informed consent was obtained from all participants. The research group received deidentified data, with the key securely stored at Statistics Sweden. The national ethical board granted ethical approval for this study (registered number 2023-04427-01).

### Variables

#### Dependent Variables

Outcomes for care recipients, measured by the perceptions of care workers, were continuity of care, participation, and reduction in loneliness and social isolation (hereafter referred to as loneliness). These indicators were measured with three questions: (1) Has the use of welfare technology contributed to the continuity of care for care recipients? (2) Has the use of welfare technology contributed to increased participation of care recipients? and (3) Has the use of welfare technology contributed to reduced loneliness and social isolation among care recipients? The response alternatives were: no, not at all; yes, to some extent; yes, to a relatively high extent; yes, to a high extent. These variables were modeled as dichotomous (no, not at all: 0; yes: 1).

The upskilling index was developed based on 2 questions: (1) Has the use of welfare technology contributed to the development of competence in care? (2) Has the use of welfare technology contributed to new competence? The response alternatives were: no, not at all; yes, to some extent; yes, to a relatively high extent; yes, to a high extent. These variables were modeled as dichotomous (no, not at all: 0; yes: 1).

#### Exposure Variables

Types of welfare technologies were measured by asking, “What kind of welfare technology do you use in your work (fill in all the options you use)?” The response options were (1) digital locks to get access to the care recipient’s home, (2) cameras or sensors for supervision, (3) digital medication (eg, medicine dispensers, robots, reminders), (4) digital support for care recipient in grocery shopping, (5) digital support for care recipient in training and activity (eg, VR, animal robots, and apps), (6) digital communications between you and care recipient (eg, video calls), (7) digital communication between you and your colleagues, and (8) digital documentation and planning (eg, using apps to register visits to care recipients or to document notes made at the visit). The Swedish National Board of Health and Welfare conducts annual data collection on types of welfare technology used in social care and municipal health care [23]. These data informed the development of response categories on types of welfare technology. Moreover, the selected welfare technologies are consistent with the findings of a recent exploratory study [24].

Training in the use of welfare technologies was measured from a single item: Have you received any special training to improve your skills in using welfare technology? The response options were (1) yes, I have received training on using a specific type of welfare technology; (2) yes, I have received training for more general technical skills related to my professional role; and (3) no, I have not received training on using welfare technology. In regression, this was modeled as a dichotomous variable (no: 0; yes: 1).

### Covariates

Age, sex, level of education, workplace (care home and home care), and work experience were the covariates. Potential covariates were selected based on a theoretical model [6], previous research [8], or possible differences in care routines at the care home and in-home care.

### Data Analysis

Characteristics of the study participants were presented in absolute and relative frequencies (Table 1). The continuous variable was reported as mean and SD. To increase the sample size (and thereby statistical power), types of technologies were grouped into 5 categories. Digital locks and cameras or sensors were combined into a category. Digital support in grocery shopping, digital support for care recipients in training and activity, and digital communications with care recipients require the care recipients to actively interact with the technology; therefore, these technologies were grouped under the category interactive technology.

**Table 1.** Characteristics of the study participants.

Characteristics	Values (N=1163)^a^
Age (years), mean (SD)	51.9 (9.6)
Sex, n (%)	
Male	80 (6.9)
Female	1083 (93.1)
Education, (n%)	
Primary or lower secondary	24 (2.1)
Upper secondary ≤2 years	459 (39.5)
Upper secondary 3 years	501 (43.1)
Postsecondary <3 years	124 (10.7)
Postsecondary ≥3 years	55 (4.7)
Workplace, n (%)	
Home care	382 (32.8)
Care home	752 (64.7)
Work experience (years), n (%)	
≥15	902 (77.6)
10‐14	146 (12.6)
<10	110 (9.5)
Welfare technologies, n (%)	
Digital locks	619 (53.2)
Cameras or sensors	387 (33.3)
Digital support in medicine	362 (31.1)
Activity and training	110 (9.5)
Digital support in grocery shopping	169 (14.5)
Digital communication between care provider and care recipient	103 (8.9)
Digital communication between colleagues	271 (23.3)
Digital documentation and planning	852 (73.3)
Not using welfare technologies	64 (5.8)
Training in the use of welfare technologies, n (%)	606 (52.1)
Continuity of care, n (%)	603 (51.8)
Patient participation, n (%)	511 (43.1)
Reduction in loneliness or social isolation, n (%)	420 (36.1)
Upskilling, n (%)	810 (69.6)

a Missing data were 2.5% for workplace and 0.4% for work experience. A total of 51 participants lacked complete information on welfare technologies, training, and dependent variables. Missing values for dependent variables were 9% for training, 11% for continuity of care, 10% for participation, and 8% for upskilling.

Logistic regression models were used to analyze the associations between independent and dependent variables. The proportion of missing data ranged from 0.4% to 11% (Table 1), with 51 participants lacking complete information on exposure and dependent variables. Given a relatively low level of missingness, we conducted a complete case analysis. Multivariate model building was done in 4 steps (Multimedia Appendix 1). At the first step, covariates age, sex, education, workplace, and work experience were entered. At the second step, types of welfare technologies were entered. At the third step, training in the use of welfare technologies was entered. At the fourth step, interaction between training in the use of welfare technologies and types of welfare technologies was entered. Only those interactions that significantly contributed to the model were considered. Results are presented as OR with 95% CI.

The deviance statistic (−2LL), Cox and Snell’s *R*^2^ (*R*^2^_CS_), and Nagelkerke *R*^2^ (*R*^2^_N_) tests were used for model building [25]. The deviance describes the unexplained variance in the model, so the smaller the value of deviance, the better the model fits the data. *R*^2^_CS_ and *R*^2^_N_ are approximations of the *R*^2^ statistic for multiple linear regression, which describes the variance in the dependent variable explained by the model. *R*^2^_CS_ and *R*^2^_N_ are calculated differently and may provide divergent estimates, thus both were included. The variance inflation factor was used to check potential multicollinearity among independent variables. The variance inflation factor of less than 10 was observed for all variables and deemed acceptable [25].

The interaction between digital documentation and planning (hereafter referred to as digital documentation) and training significantly contributed to the regression model (Multimedia Appendix 1). To evaluate the interaction effect of digital documentation and training, we predicted the probabilities for the outcome using the full model, setting the values of nonfocal variables to their mean values, and visualized the results with 95% CI. A significance level <.05 was used. The final multivariate models, including associations between independent and dependent variables, are presented in the Results section, whereas interaction effects are illustrated as figures. SPSS V.29 (IBM Corp) for Windows and Statistical software R version 4.4.1 (R Development Core Team) were used to conduct all analyses.

## Results

### Characteristics of the Study Participants

A sample of 5000 individuals received an invitation, of whom 1163 participated in the study. The mean age of the study participants was 51.9 (SD 9.6) years, and 1083 out of 1163 (93%) participants were women (Table 1). Only 24 out of 1163 (2.1%) participants had primary or lower secondary education. However, upper secondary education was common among the participants, of whom 459 (39.5%) completed up to 2 years and 501 (43.1%) completed 3 years. Among the 1163 participants, 179 (15%) had postsecondary education. Of the 1163, 752 (64.7%) participants worked in care homes, and 382 (32.8%) worked in home care. The work experience of at least 15 years was reported by 902 out of 1163 participants (77.6%); 146 participants (12.6%) had 10‐14 years, and 110 participants (9.5%) had less than 10 years of experience. Among the 1163 participants, 852 reported use of digital documentation (73.3%) and 619 reported use of digital locks (53.2%). Of 1163 participants, 110 (9.5%) indicated that they used technologies for activity or training, and 103 participants (8.9%) reported digital communication with care recipients. A total of 606 participants out of 1163 (52.1%) had been trained in the use of welfare technologies. In response to the question regarding the use of welfare technologies, 603 out of 1163 (51.8%) participants were positive regarding continuity of care, 511 participants (43.1%) regarding participation, 420 (36.1%) regarding reduction in loneliness, and 810 (69.6%) regarding upskilling (Table 1).

### Continuity of Care

In the multivariate model, interactive technologies were statistically significantly associated with the higher odds of continuity of care (OR 1.58, 95% CI 1.15‐2.18) (Table 2). In contrast, digital locks and cameras or sensors (OR 0.79, 95% CI 0.58‐1.08), digital support in medicine (OR 1.29, 95% CI 0.96‐1.75), digital documentation (OR 1.33, 95% CI 0.95‐1.85), and digital communication with colleagues (OR 0.99, 95% CI 0.72‐1.38) were the types of welfare technologies with no significant association with continuity of care. However, training in the use of welfare technology was significantly associated with continuity of care (OR 2.02, 95% CI 1.53‐2.66).

**Figure 1.**
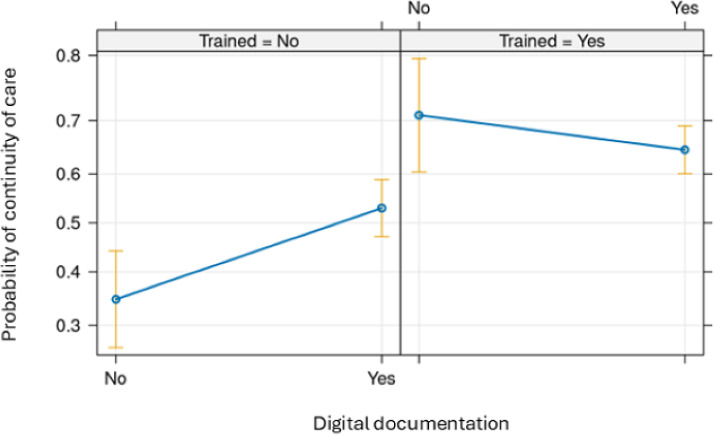
Predicted probability of continuity of care as a function of digital documentation and training in welfare technology.

The interaction analysis has shown that the participants who did not have training but used digital documentation had a higher probability of positive perceptions regarding continuity of care. Although perceptions on continuity were positive among participants who had training, the use of digital documentation had no significant effect (Figure 1).

**Table 2.** Models for binary logistic regression exploring associations between welfare technologies, training, and potential outcomes for frontline care workers and care recipients.

Variables	Continuity of care	Participation	Reduce loneliness	Upskilling
Covariates, OR^a^ (95% CI)				
Age (years)	1.02 (1.00‐1.03)^b^	1.00 (0.99‐1.02)	0.99 (0.98‐1.02)	1.03 (1.01‐1.05)^b^
Female	0.58 (0.32‐1.02)	1.17 (0.68‐2.02)	0.98 (0.57‐1.69)	0.48 (0.23‐0.99)^b^
Workplace care home	1.07 (0.79‐1.43)	1.03 (0.77‐1.38)	1.31 (0.97‐1.76)	0.97 (0.67‐1.39)
Work experience (years), OR (95% CI)				
≥15	Ref.^c^	Ref.	Ref.	Ref.
10‐14	1.86 (1.18‐2.91)^b^	1.63 (1.06‐2.49)^b^	1.61 (1.06‐2.46)^b^	1.46 (0.85‐2.50)
<10	2.25 (1.28‐3.96)^b^	1.39 (0.82‐2.38)	1.96 (1.16‐3.31)^b^	1.75 (0.89‐3.44)
Education, OR (95% CI)				
Upper secondary <2 years	Ref.	Ref.	Ref.	Ref.
Upper secondary 3 years	0.94 (0.69‐1.28)	0.88 (0.66‐1.20)	0.96 (0.71‐1.31)	1.32 (0.91‐1.91)
Postsecondary	0.98 (0.66‐1.48)	0.90 (0.61‐1.33)	0.93 (0.62‐1.38)	1.24 (0.75‐2.05)
Types of technologies, OR (95% CI)				
Digital locks and cameras or sensors	0.79 (0.58‐1.08)	0.62 (0.46‐0.84)^b^	0.77 (0.57‐1.05)	1.05 (0.73‐1.49)
Digital support in medicine	1.29 (0.96‐1.75)	1.17 (0.87‐1.56)	1.13 (0.83‐1.51)	1.59 (1.08‐2.35)^b^
Interactive technologies	1.58 (1.15‐2.18)^b^	2.01 (1.48‐2.74)^b^	1.92 (1.41‐2.62)^b^	2.44 (1.58‐3.79)^b^
Digital documentation	1.33 (0.95‐1.85)	0.69 (0.49‐0.96)^b^	0.73 (0.52‐1.02)	2.08 (1.45‐2.99)^b^
Digital communication with colleagues	0.99 (0.72‐1.38)	1.01 (0.73‐1.38)	0.82 (0.59‐1.14)	1.28 (0.83‐1.97)
Training, OR (95% CI)				
Training in the use of welfare technologies	2.02 (1.53‐2.66)^b^	1.91 (1.45‐2.51)^b^	1.74 (1.31‐2.30)^b^	4.59 (3.28‐6.42)^b^
−2LL^d^	1260.596	1297.035	1267.204	938.650
*R* ^2^_CS_ ^e^	0.075	0.069	0.058	0.175
Nagelkerke *R*^2^_N_^f^	0.100	0.092	0.079	0.260

a OR: odds ratio.

b Statistical significance at the *P*<.05 level.

c Ref.: reference.

d −2LL: deviance.

e *R*2_CS_: Cox and Snell *R*2.

f *R*2_N_: Nagelkerke *R*2.

### Participation of Care Recipients

In the multivariate model, use of digital locks and cameras or sensors (OR 0.62, 95% CI 0.46‐0.84) and digital documentation (OR 0.69, 95% CI 0.49‐0.96) were significantly associated with a lower OR of participation (Table 2). Conversely, the use of interactive technologies was significantly associated with participation (OR 2.01, 95% CI 1.48‐2.74). The types of welfare technologies that were not significantly associated with participation were digital support in medicine (OR 1.17, 95% CI 0.87‐1.56) and digital communication with colleagues (OR 1.01, 95% CI 0.73‐1.38). Nevertheless, there was a significant association between training in the use of welfare technology and participation (OR 1.91, 95% CI 1.45‐2.51).

**Figure 2.**
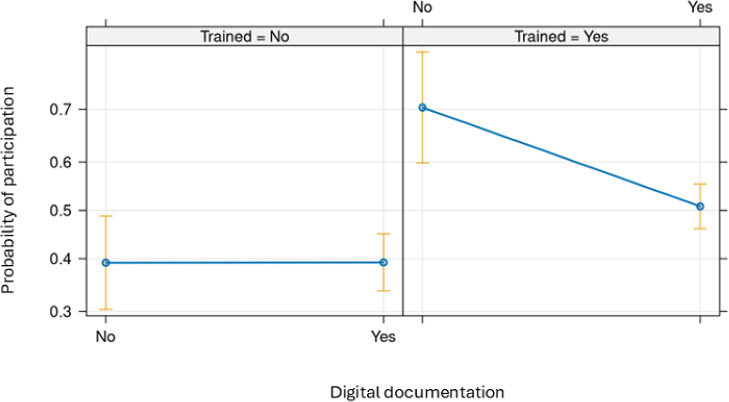
Predicted probability of participation as a function of digital documentation and training in welfare technology.

Training was positively associated with perceptions of participation; however, this effect was attenuated when combined with digital documentation. Among those who had no training, digital documentation had not shown any effect on participation, while among care workers who had training, it was associated with a decreased probability of positive perceptions (Figure 2).

### Reduction in Loneliness

Care workers’ perception of addressing loneliness among care recipients has shown a statistically significant association between the use of interactive technologies and reduced loneliness among care recipients (OR 1.92, 95% CI 1.41‐2.62) (Table 2). All other technologies included in this study had no significant association with loneliness such as digital locks and cameras or sensors (OR 0.77, 95% CI 0.57‐1.05), digital support in medicine (OR 1.13, 95% CI 0.83‐1.51), digital documentation (OR 0.73, 95% CI 0.52‐1.02), and digital communication with colleagues (OR 0.82, 95% CI 0.59‐1.14). Care workers who received training in welfare technology had positive perceptions of its use for the reduction in loneliness of the care recipients (OR 1.74, 95% CI 1.31‐2.30).

**Figure 3.**
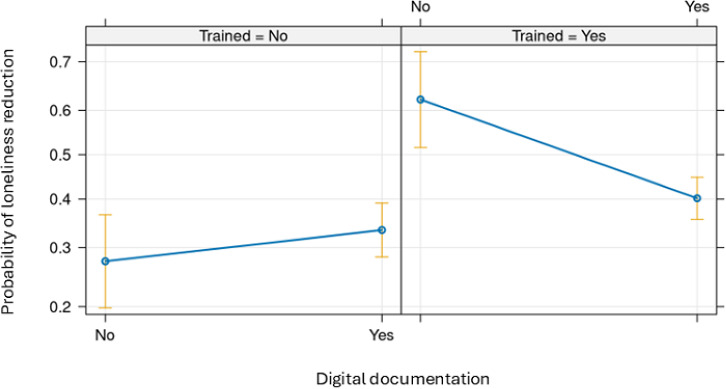
Predicted probability of reduction in loneliness as a function of digital documentation and training in welfare technology.

Among those who had training, digital documentation has shown a significantly decreased probability of positive perception on reduction in loneliness (Figure 3). However, the association between digital documentation and the probability of outcome was not significant in the group with no training.

### Upskilling

**Figure 4.**
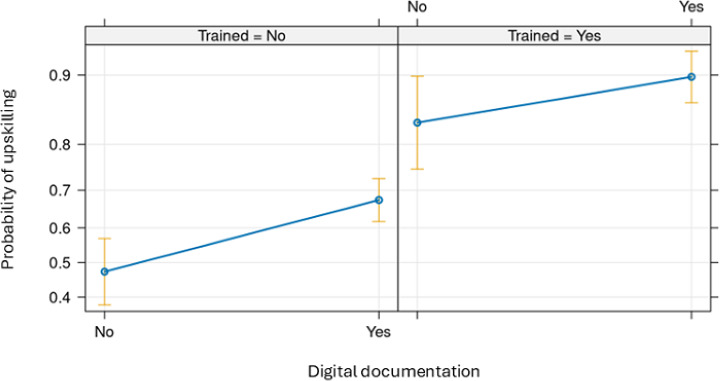
Predicted probability of upskilling as a function of digital documentation and training in welfare technology.

In the multivariate model (Table 2), types of welfare technologies significantly associated with higher ORs of upskilling among care workers were digital support in medicine (OR 1.59, 95% CI 1.08‐2.35), use of interactive technologies (OR 2.44, 95% CI 1.58‐3.79), and digital documentation (OR 2.08, 95% CI 1.45‐2.99), whereas digital locks and cameras or sensors (OR 1.05, 95% CI 0.73‐1.49) and digital communication with colleagues (OR 1.28, 95% CI 0.83‐1.97) were the technologies not significantly associated with upskilling. Nevertheless, training in the use of welfare technology was significantly associated with upskilling among care workers (OR 4.59, 95% CI 3.28‐6.42) (Table 2). Digital documentation has shown a higher probability of upskilling, but the effect was significant only in the group that did not have training (Figure 4).

## Discussion

### Principal Findings

Based on the perceptions of assistant nurses, this study explored associations between types of welfare technologies and outcomes for care recipients as well as for care workers and whether training in the use of welfare technologies was associated with these outcomes. We found that the use of interactive technologies was associated with the continuity of care, participation, and reduction in loneliness among care recipients, and upskilling of frontline care workers. However, not all types of technologies had similar effects. For example, the use of digital documentation was associated with upskilling but negatively associated with participation. In addition, the use of digital locks and cameras or sensors was negatively associated with participation only. Furthermore, while digital support in medicine was associated with upskilling among care workers, no significant associations were seen for care recipients. Training in the use of welfare technologies was independently associated with all the dependent variables, yet the interaction of training and use of digital documentation had mixed effects.

### Comparison With Prior Research

This study’s findings on interactive technologies are broadly in line with those of studies highlighting the importance of technologies supporting communication to improve quality of care [26], participation [24], and to reduce loneliness among care recipients [10]. Interactive technologies require care recipients to take an active role; for example, training, grocery shopping, video conferencing, and contributing to the involvement and empowerment of care recipients. Care workers perceive that such technologies provide a range of options that can be customized to meet the specific needs or preferences of care recipients. In addition, these technologies facilitate joint activities between care workers and care recipients while also having an important role in keeping care recipients engaged [24]. Hence, technologies supporting a sense of connection and time spent with care recipients are perceived as important by formal and informal caregivers for improving quality of care, job satisfaction, and acceptance of technology [5,9,26].

The introduction of technology in the workplace changes job design, fostering the acquisition of new skills [18]. Care workers perform various combinations of tasks, using different types of welfare technologies, each requiring different levels of skill. This diversity makes it difficult to establish a clear reference point for skill development [17]. Moreover, the lack of studies on types of technologies and upskilling limits our ability to make comparisons with previous research. Our study shows a positive association between types of welfare technologies and upskilling. However, the use of digital locks and communication tools among colleagues did not reach statistical significance. These technologies do not demand high-level skills. Furthermore, care workers often perceive digital locks as tools of convenience [24] and likely explain our findings.

The associations between training in the use of welfare technologies and improved outcomes contribute to the empirical evidence supporting the idea that training can contribute to the positive experiences with using technology [9,11] and skill development among care workers [18]. Training endows essential skills in the use of technology and enhances critical reasoning in relation to technology use and quality of care, hence potentially contributing to upskilling.

Previous research has shown that digital documentation enables faster access to necessary information that can help reduce errors and promote collaborative care [11,14]. These attributes of digital documentation contribute to informational continuity according to the views of Swedish primary health care nurses [14], and this is consistent with our findings on continuity, but only among those who did not have training and use digital documentation. Nevertheless, multidimensional aspects of the concept of continuity of care and critical reasoning likely explain a nonsignificant effect of digital documentation on the probability of continuity among those who received training. For example, digital records may not ensure interrelation (patient and single provider) continuity of care. Moreover, the administrative character of digital documentation does not align with the fundamental value of patient-provider interaction in nursing care [9,14] and likely explains our findings on the combined effects of training and use of digital documentation on a lower likelihood of participation and in addressing loneliness among care recipients. An exploratory qualitative study can offer insights into the combined effects of training and digital documentation on diverse perceptions of care delivery outcomes.

Assistant nurses sometimes perceive digital documentation as time-consuming and monotonous [24]. Such repetitive work is often perceived as low in complexity and therefore seen as offering limited opportunities for upskilling [17]. However, the interaction analyses have shown a positive association between digital documentation and upskilling in our study. In repetitive work, the repeated use of specific skills can lead to greater fluency and precision, suggesting that such tasks may still offer learning potential [18].

### Potential Implications

Training provision improves skills and fosters capacities to adapt to workplace innovations [17,18]. In line with this understanding, our findings on training and improved outcomes suggest that investing resources to promote training can reinforce positive experiences, thereby sustaining social care practices.

This study shows that care workers hold more positive perceptions toward technologies that promote the active involvement of care recipients. Nevertheless, consistent with previous research, such technologies remain among the least commonly implemented in older adult care settings [4]. Their broader adoption could play an important role in enhancing care recipient engagement and social participation. To fully understand the potential of these technologies, future research should incorporate the perspectives and lived experiences of care recipients themselves.

### Strengths and Limitations

A strength of this study is that it is based on a survey including a randomized national sample exploring the potential benefits of welfare technology for care recipients as well as for care workers. The study sample included both public and private providers.

This study has a few limitations, including that the cross-sectional study design limits the ability to draw a temporal relation between the exposure and outcome. The low response rate (23%), particularly among younger age groups (aged <44 years), may pose a risk of selection bias, limiting the generalizability of our findings. There is no consensus on the definition of continuity of care and participation, which may lead to slightly different interpretations of concepts. As noted, this research project and the questionnaire are based on an earlier project. However, questions related to welfare technology were new, which can influence internal validity. National reports and experiences of the research team informed the development of the questionnaire. Moreover, studies in the Nordic context have used a similar approach [4]. Therefore, the problem is minimal.

Participants with missing data on welfare technology may have different opinions; however, they are few (n=51). The survey was not limited to welfare technology, and the items related to technology were placed at the end. This is likely that the participants have other reasons to stop the survey, for example, tiredness. Receipt of training was measured by a single item that is limited to cover components of training. Moreover, local variations can be expected in the content and form of training. Finally, the amount of variance our models explained in their respective outcomes was not substantial; this was particularly the case for outcomes for care recipients. This is perhaps not surprising given the many factors that might potentially influence outcomes for older adults with complex care needs.

### Conclusions

This study contributes by identifying how the type of welfare technology and training has an impact. We found that the potential outcomes of welfare technology in social care for older adults can vary with the types of technologies used. Specifically, care workers hold positive perceptions that the use of interactive technologies is associated with the continuity of care, participation, and reduction in loneliness among care recipients, suggesting the importance of patient-provider interaction. Despite the benefits of welfare technology, the effects can be negative, as shown by the findings on the use of digital documentation, digital locks and cameras or sensors, and the lower likelihood of participation. Nevertheless, care workers hold favorable views regarding the use of welfare technologies contributing positively to their skill development.

Training in the use of welfare technologies was positively associated with the perceived usefulness of these technologies in continuity of care, participation, reduction in loneliness of care recipients, and upskilling of care workers. While training had a positive impact, the effects of digital documentation were mixed in relation to training. Notably, participants who had not received any training but used digital documentation reported favorable views on continuity and upskilling, whereas participants with training expressed concerns about participation and addressing loneliness. Future research should explore how specific training components interact with digital documentation to better understand their combined influence on care outcomes.

## Supplementary material

10.2196/65641 Multimedia Appendix 1 Model building for binary logistic regression exploring associations between welfare technologies, training, and potential outcomes for frontline care workers and care recipients

## Abbreviations

OR: odds ratio
*R* ^2^_CS_: Cox and Snell *R*^2^
*R* ^2^_N_: Nagelkerke *R*^2^

## References

[1] Härkönen H, Lakoma S, Verho A (2024). Impact of digital services on healthcare and social welfare: an umbrella review. Int J Nurs Stud.

[2] Frennert S, Baudin K (2021). The concept of welfare technology in Swedish municipal eldercare. Disabil Rehabil.

[3] Kamp A, Obstfelder A, Andersson K (2019). Welfare technologies in care work. Nord J Work Life Stud.

[4] Rostad HM, Stokke R (2021). Integrating welfare technology in long-term care services: nationwide cross-sectional survey study. J Med Internet Res.

[5] Kaihlanen AM, Laukka E, Nadav J (2023). The effects of digitalisation on health and social care work: a qualitative descriptive study of the perceptions of professionals and managers. BMC Health Serv Res.

[6] Venkatesh V, Morris M, Davis G, Davis F (2003). User acceptance of information technology: toward a unified view. MIS Q.

[7] Davis FD, Bagozzi RP, Warshaw PR (1989). User acceptance of computer technology: a comparison of two theoretical models. Manage Sci.

[8] Barchielli C, Marullo C, Bonciani M, Vainieri M (2021). Nurses and the acceptance of innovations in technology-intensive contexts: the need for tailored management strategies. BMC Health Serv Res.

[9] Jedwab RM, Manias E, Redley B, Dobroff N, Hutchinson AM (2023). Impacts of technology implementation on nurses’ work motivation, engagement, satisfaction and well-being: a realist review. J Clin Nurs.

[10] Tian YJA, Felber NA, Pageau F, Schwab DR, Wangmo T (2024). Benefits and barriers associated with the use of smart home health technologies in the care of older persons: a systematic review. BMC Geriatr.

[11] Wosny M, Strasser LM, Hastings J (2023). Experience of health care professionals using digital tools in the hospital: qualitative systematic review. JMIR Hum Factors.

[12] Haggerty JL, Reid RJ, Freeman GK, Starfield BH, Adair CE, McKendry R (2003). Continuity of care: a multidisciplinary review. BMJ.

[13] (2022). Vård och omsorg om äldre: lägesrapport 2022 [Report in Swedish]. https://www.socialstyrelsen.se/publikationer/vard-och-omsorg-for-aldre--lagesrapport-2022-2022-3-7791/.

[14] Hellzén O, Kjällman Alm A, Holmström Rising M (2022). Primary healthcare nurses’ views on digital healthcare communication and continuity of care: a deductive and inductive content analysis. Nurs Rep.

[15] Zander V, Johansson-Pajala RM, Gustafsson C (2020). Methods to evaluate perspectives of safety, independence, activity, and participation in older persons using welfare technology. a systematic review. Disabil Rehabil Assist Technol.

[16] Grey E, Baber F, Corbett E, Ellis D, Gillison F, Barnett J (2024). The use of technology to address loneliness and social isolation among older adults: the role of social care providers. BMC Public Health.

[17] Martinaitis Ž, Christenko A, Antanavičius J (2021). Upskilling, deskilling or polarisation? Evidence on change in skills in Europe. Work, Employment and Society.

[18] (2016). Job design and skill developments in the workplace. https://www.econstor.eu/bitstream/10419/147893/1/dp10207.pdf.

[19] Nilsen ER, Dugstad J, Eide H, Gullslett MK, Eide T (2016). Exploring resistance to implementation of welfare technology in municipal healthcare services - a longitudinal case study. BMC Health Serv Res.

[20] Konttila J, Siira H, Kyngäs H (2019). Healthcare professionals’ competence in digitalisation: a systematic review. J Clin Nurs.

[21] Ladds E, Khan M, Moore L, Kalin A, Greenhalgh T (2023). The impact of remote care approaches on continuity in primary care: a mixed-studies systematic review. Br J Gen Pract.

[22] Dellve L, Lagerström M, Hagberg M (2003). Work-system risk factors for permanent work disability among home-care workers: a case-control study. Int Arch Occup Environ Health.

[23] (2023). E-hälsa och välfärdsteknik i kommunerna 2023: uppföljning av den digitala utvecklingen i socialtjänst och kommunal hälso- och sjukvård [Report in Swedish]. https://www.socialstyrelsen.se/contentassets/26ae5fe4c8864951b0926a0c99decf34/2023-5-8549.pdf.

[24] Frennert S, Skagert K, Williamsson A (2024). It is a matter of convenience: why welfare technologies have become domesticated in Swedish eldercare. BMC Health Serv Res.

[25] Field A (2013). Discovering Statistics Using IBM SPSS Statistics.

[26] Leslie M, Gray RP, Khayatzadeh-Mahani A (2021). What is “care quality” and can it be improved by information and communication technology? A typology of family caregivers’ perspectives. Scand J Caring Sci.

