# Supplementary material for: Assessing the Use of Welfare Technology in Social Care for Older Adults Through Assistant Nurses’ Perceptions of Upskilling and Care Delivery Outcomes: Cross-Sectional Study
Source: JMIR Nurs. 2025 Aug 26;8:e65641. doi: 10.2196/65641 (PMC12379745; doi:10.2196/65641)
Supplement: Multimedia Appendix 1 [file nursing-v8-e65641-s001.docx]

**Table S1.** Models for binary logistic regression of factors associated with continuity of care (n=981)

| Variables | **Model 1** | **Model 2** | **Model Final** | **Model 4** |
| --- | --- | --- | --- | --- |
|  | OR (95% CI) | OR (95% CI) | OR (95% CI) | OR (95% CI) |
|  |  |  |  |  |
| **Covariates** |  |  |  |  |
| Age | 1.02 (1.00-1.03) | 1.02 (1.00-1.04) | 1.02 (1.00-1.03) | 1.02 (1.00-1.03) |
| Women | 0.69 (0.39-1.19) | 0.59 (0.33-1.03) | 0.58 (0.32-1.02) | 0.55 (0.31-0.99) |
| Workplace care home | 1.05 (0.80-1.38) | 1.12 (0.84-1.49) | 1.07 (0.79-1.43) | 1.07 (0.79-1.44) |
| Work experience |  |  |  |  |
| ≥ 15 years | Ref. | Ref. | Ref. | Ref. |
| 10-14 years | 1.83 (1.18-2.82) | 1.85 (1.19-2.90) | 1.86 (1.18-2.91) | 1.79 (1.14-2.81) |
| <10 years | 2.27 (1.31-3.93) | 2.30 (1.32-4.04) | 2.25 (1.28-3.96) | 2.27 (1.28-4.00) |
| Education |  |  |  |  |
| Lower or upper-secondary <2years | Ref. | Ref. | Ref. | Ref. |
| Upper-secondary 3years | 0.89 (0.66-1.19) | 0.93 (0.68-1.25) | 0.94 (0.69-1.28) | 0.94 (0.69-1.28) |
| Post secondary | 1.05 (0.71-1.55) | 1.05 (0.70-1.56) | 0.98 (0.66-1.48) | 0.99 (0.67-1.49) |
| **Types of technologies** |  |  |  |  |
| Digital locks & Cameras/sensors |  | 0.86 (0.64-1.16) | 0.79 (0.58-1.08) | 0.78 (0.57-1.07) |
| Digital support in medicine |  | 1.46 (1.09-1.96) | 1.29 (0.96-1.75) | 1.32 (0.98-1.79) |
| Interactive technologies |  | 1.69 (1.23-2.31) | 1.58 (1.15-2.18) | 1.57 (1.14-2.16) |
| Digital documentation |  | 1.44 (1.04-2.00) | 1.33 (0.95-1.85) | 2.13 (1.34-3.37) |
| Digital communication with colleagues |  | 1.03 (0.75-1.42) | 0.99 (0.72-1.38) | 1.00 (0.72-1.39) |
| **Training** |  |  |  |  |
| Training in the use of welfare technologies |  |  | 2.02 (1.53-2.66) | 4.59 (2.49-8.47) |
| Training*Digital documentation |  |  |  | 0.35 (0.18-0.69) |
|  |  |  |  |  |
| Deviance (-2LL) | 1317.157 | 1285.512 | 1260.596 | 1251.244 |
| Cox and Snell R^2^_CS_ | 0.020 | 0.051 | 0.075 | 0.083 |
| Nagelkerke R^2^_N_ | 0.026 | 0.068 | 0.100 | 0.112 |
| p-value | 0.007 | <0.001 | <0.001 | 0.002 |

**Table S2.** Models for binary logistic regression of factors in relation to welfare technologies associated with participation (n=988)

| Variables | **Model 1** | **Model 2** | **Model Final** | **Model 4** |
| --- | --- | --- | --- | --- |
|  | OR (95% CI) | OR (95% CI) | OR (95% CI) | OR (95% CI) |
|  |  |  |  |  |
| **Covariates** |  |  |  |  |
| Age | 1.00 (0.99-1.02) | 1.01 (0.99-1.02) | 1.00 (0.99-1.02) | 1.00 (0.99-1.02) |
| Women | 1.26 (0.75-2.12) | 1.18 (0.69-2.01) | 1.17 (0.68-2.02) | 1.15 (0.67-1.99) |
| Workplace care home | 1.08 (0.82-1.40) | 1.08 (0.81-1.44) | 1.03 (0.77-1.38) | 1.03 (0.77-1.38) |
| Work experience |  |  |  |  |
| ≥ 15 years | Ref. | Ref. | Ref. | Ref. |
| 10-14 years | 1.73 (1.15-2.62) | 1.63 (1.07-2.49) | 1.63 (1.06-2.49) | 1.58 (1.03-2.43) |
| <10 years | 1.49 (0.89-2.48) | 1.43 (0.85-2.42) | 1.39 (0.82-2.38) | 1.41 (0.83-2.39) |
| Education |  |  |  |  |
| Lower or Upper secondary <2years | Ref. | Ref. | Ref. | Ref. |
| Upper secondary 3 years | 0.82 (0.61-1.10) | 0.88 (0.65-1.18) | 0.88 (0.66-1.20) | 0.89 (0.66-1.21) |
| Post secondary | 0.89 (0.62-1.31) | 0.95 (0.64-1.40) | 0.90 (0.61-1.33) | 0.91 (0.61-1.35) |
| **Types of technologies** |  |  |  |  |
| Digital locks & Cameras/sensors |  | 0.67 (0.49-0.89) | 0.62 (0.46-0.84) | 0.62 (0.46-0.83) |
| Digital support in medicine |  | 1.30 (0.98-1.73) | 1.17 (0.87-1.56) | 1.18 (0.88-1.58) |
| Interactive technologies |  | 2.13 (1.57-2.88) | 2.01 (1.48-2.74) | 2.00 (1.47-2.72) |
| Digital documentation |  | 0.75 (0.54-1.04) | 0.69 (0.49-0.96) | 1.00 (0.64-1.58) |
| Digital communication with colleagues |  | 1.04 (0.76-1.43) | 1.01 (0.73-1.38) | 1.01 (0.74-1.39) |
| **Training** |  |  |  |  |
| Training in the use of welfare technologies |  |  | 1.91 (1.45-2.51) | 3.68 (2.02-6.71) |
| Training*Digital documentation |  |  |  | 0.43 (0.22-0.85) |
|  |  |  |  |  |
| Deviance (-2LL) | 1357.008 | 1318.466 | 1297.035 | 1290.949 |
| Cox and Snell R^2^_CS_ | 0.011 | 0.048 | 0.069 | 0.075 |
| Nagelkerke R^2^_N_ | 0.014 | 0.065 | 0.092 | 0.099 |
| p-value | 0.162 | <0.001 | <0.001 | 0.014 |

**Table S3.** Models for binary logistic regression of factors in relation to welfare technologies associated to reduce loneliness or social isolation among care recipients (N=992)

| Variables | **Model 1** | **Model 2** | **Model Final** | **Model 4** |
| --- | --- | --- | --- | --- |
|  | OR (95% CI) | OR (95% CI) | OR (95% CI) | OR (95% CI) |
|  |  |  |  |  |
| **Covariates** |  |  |  |  |
| Age | 0.99 (0.98-1.02) | 1.00 (0.98-1.02) | 0.99 (0.98-1.02) | 0.99 (0.98-1.02) |
| Women | 1.07 (0.63-1.82) | 0.99 (0.58-1.69) | 0.98 (0.57-1.69) | 0.95 (0.55-1.65) |
| Workplace care home | 1.29 (0.98-1.70) | 1.35 (1.01-1.82) | 1.31 (0.97-1.76) | 1.31 (0.97-1.77) |
| Work experience |  |  |  |  |
| ≥ 15 years | Ref. | Ref. | Ref. | Ref. |
| 10-14 years | 1.73 (1.15-2.60) | 1.63 (1.08-2.47) | 1.61 (1.06-2.46) | 1.56 (1.02-2.38) |
| <10 years | 2.06 (1.24-3.44) | 1.99 (1.18-3.36) | 1.96 (1.16-3.31) | 1.97 (1.16-3.35) |
| Education |  |  |  |  |
| Lower or Upper secondary <2years | Ref. | Ref. | Ref. | Ref. |
| Upper secondary 3 years | 0.89 (0.66-1.21) | 0.96 (0.70-1.29) | 0.96 (0.71-1.31) | 0.97 (0.71-1.32) |
| Post secondary | 0.92 (0.63-1.35) | 0.97 (0.65-1.44) | 0.93 (0.62-1.38) | 0.94 (0.63-1.40) |
| **Types of technologies** |  |  |  |  |
| Digital locks & Cameras/sensors |  | 0.82 (0.61-1.10) | 0.77 (0.57-1.05) | 0.76 (0.56-1.04) |
| Digital support in medicine |  | 1.23 (0.92-1.65) | 1.13 (0.83-1.51) | 1.14 (0.85-1.54) |
| Interactive technologies |  | 2.02 (1.49-2.74) | 1.92 (1.41-2.62) | 1.92 (1.41-2.61) |
| Digital documentation |  | 0.79 (0.57-1.09) | 0.73 (0.52-1.02) | 1.33 (0.82-2.17) |
| Digital communication with colleagues |  | 0.85 (0.62-1.17) | 0.82 (0.59-1.14) | 0.83 (0.60-1.15) |
| **Training** |  |  |  |  |
| Training in the use of welfare technologies |  |  | 1.74 (1.31-2.30) | 4.37 (2.39-8.01) |
| Training*Digital documentation |  |  |  | 0.30 (0.16-0.60) |
|  |  |  |  |  |
| Deviance (-2LL) | 1308.784 | 1282.102 | 1267.204 | 1254.999 |
| Cox and Snell R^2^_CS_ | 0.018 | 0.044 | 0.058 | 0.070 |
| Nagelkerke R^2^_N_ | 0.025 | 0.060 | 0.079 | 0.095 |
| p-value | 0.011 | <0.001 | <0.001 | <0.001 |

**Table S4.** Models for binary logistic regression of factors in relation to welfare technologies associated with competence development among frontline care providers (N=1016)

| Variables | **Model 1** | **Model 2** | **Model Final** | **Model 4** |
| --- | --- | --- | --- | --- |
|  | OR (95% CI) | OR (95% CI) | OR (95% CI) | OR (95% CI) |
|  |  |  |  |  |
| **Covariates** |  |  |  |  |
| Age | 1.02 (1.01-1.04) | 1.04 (1.02-1.06) | 1.03 (1.01-1.05) | 1.03 (1.01-1.05) |
| Women | 0.71 (0.37-1.38) | 0.52 (0.26-1.05) | 0.48 (0.23-0.99) | 0.47 (0.23-0.98) |
| Workplace care home | 0.84 (0.62-1.15) | 1.08 (0.78-1.52) | 0.97 (0.67-1.39) | 0.97 (0.67-1.39) |
| Work experience |  |  |  |  |
| ≥ 15 years | Ref. | Ref. | Ref. | Ref. |
| 10-14 years | 1.38 (0.85-2.24) | 1.51 (0.90-2.53) | 1.46 (0.85-2.50) | 1.45 (0.84-2.48) |
| <10 years | 1.74 (0.93-3.26) | 1.94 (1.00-3.74) | 1.75 (0.89-3.44) | 1.76 (0.89-3.46) |
| Education |  |  |  |  |
| Lower or Upper secondary <2years | Ref. | Ref. | Ref. | Ref. |
| Upper secondary 3 years | 1.17 (0.84-1.63) | 1.26 (0.88-1.78) | 1.32 (0.91-1.91) | 1.32 (0.92-1.91) |
| Post secondary | 1.44 (0.91-2.26) | 1.39 (0.86-2.24) | 1.24 (0.75-2.05) | 1.24 (0.75-2.06) |
| **Types of technologies** |  |  |  |  |
| Digital locks & Cameras/sensors |  | 1.23 (0.88-1.72) | 1.05 (0.73-1.49) | 1.04 (0.73-1.49) |
| Digital support in medicine |  | 2.02 (1.39-2.93) | 1.59 (1.08-2.35) | 1.60 (1.08-2.36) |
| Interactive technologies |  | 2.76 (1.81-4.21) | 2.44 (1.58-3.79) | 2.44 (1.57-3.78) |
| Digital documentation |  | 2.33 (1.65-3.29) | 2.08 (1.45-2.99) | 2.32 (1.48-3.64) |
| Digital communication with colleagues |  | 1.37 (0.91-2.07) | 1.28 (0.83-1.97) | 1.28 (0.83-1.96) |
| **Training** |  |  |  |  |
| Training in the use of welfare technologies |  |  | 4.59 (3.28-6.42) | 5.73 (2.98-11.03) |
| Training*Digital documentation |  |  |  | 0.74 (0.35-1.57) |
|  |  |  |  |  |
| Deviance (-2LL) | 1120.909 | 1024.415 | 938.650 | 938.023 |
| Cox and Snell R^2^_CS_ | 0.013 | 0.102 | 0.175 | 0.175 |
| Nagelkerke R^2^_N_ | 0.019 | 0.152 | 0.260 | 0.261 |
| p-value | 0.075 | <0.001 | <0.001 | 0.429 |
